# Risk factors and clinical outcomes of extubation failure in very early preterm infants: a single-center cohort study

**DOI:** 10.1186/s12887-023-03833-5

**Published:** 2023-01-21

**Authors:** Su Jeong Park, Mi Hye Bae, Mun Hui Jeong, Seong Hee Jeong, NaRae Lee, Shin Yun Byun, Kyung Hee Park

**Affiliations:** 1 Department of Pediatrics, Pusan National University Hospital, Pusan National University School of Medicine, Busan, Korea; 2 Department of Pediatrics, Pusan National University Children’s Hospital, Pusan National University School of Medicine, Yangsan, Korea

**Keywords:** Very early preterm infant, Mechanical ventilation, Extubation failure, Risk factors, Respiratory severity score

## Abstract

**Background:**

Early extubation success (ES) in preterm infants may reduce various mechanical ventilation-associated complications; however, extubation failure (EF) can cause adverse short- and long-term outcomes. Therefore, the present study aimed to identify differences in risk factors and clinical outcomes between ES and EF in very early preterm infants.

**Methods:**

This retrospective study was conducted between January 2017 and December 2021. Premature infants born at 32 weeks’ gestational age in whom extubation had failed at least once were assigned to the EF group. Successfully extubated patients with a similar gestational age and birth weight as those in the EF group were assigned to the ES group. EF was defined as the need for re-intubation within 120 h of extubation. Various variables were compared between groups.

**Results:**

The EF rate in this study was 18.6% (24/129), and approximately 80% of patients with EF required re-intubation within 90.17 h. In the ES group, there was less use of inotropes within 7 days of life (12 [63.2%] vs. 22 [91.7%], *p* = 0.022), a lower respiratory severity score (RSS) at 1 and 4 weeks (1.72 vs. 2.5, *p* = 0.026; 1.73 vs. 2.92, *p* = 0.010), and a faster time to reach full feeding (18.7 vs. 29.7, *p* = 0.020). There was a higher severity of bronchopulmonary dysplasia BPD (3 [15.8%] vs. 14 [58.3%], *p* = 0.018), longer duration of oxygen supply (66.5 vs. 92.9, *p* = 0.042), and higher corrected age at discharge (39.6 vs. 42.5, *p* = 0.043) in the EF group. The cutoff value, sensitivity, and specificity of the respiratory severity score (RSS) at 1 week were 1.98, 0.71, and 0.42, respectively, and the cutoff value, sensitivity, and specificity of RSS at 4 weeks were 2.22, 0.67, and 0.47, respectively.

**Conclusions:**

EF caused adverse short-term outcomes such as a higher BPD severity and longer hospital stay. Therefore, extubation in very early preterm infants should be carefully evaluated. Using inotropes, feeding, and RSS at 1 week of age can help predict extubation success.

**Supplementary Information:**

The online version contains supplementary material available at 10.1186/s12887-023-03833-5.

## Background

Most very low birth weight infants born at < 1,500 g require mechanical ventilation (MV) to maintain oxygenation and ventilation [1]. However, prolonged MV may have various adverse effects, such as upper airway injury, ventilator-associated pneumonia, and bronchopulmonary dysplasia (BPD) [2, 3]. Therefore, early extubation after MV might reduce the risk of complications. However, definite guidelines are lacking for extubation in preterm infants, and physicians must decide the timing and method of extubation according to their experience. As a result, the rate of extubation failure (EF) is up to 42% according to previous reports [4, 5]. Preterm infants who endure intubation due to EF often experience adverse events such as airway injury, bradycardia, and oxygen desaturation. Furthermore, EF is associated with the risk of intraventricular hemorrhage (IVH), BPD, and death [5, 6]. Therefore, it is important to extubate early and decrease the EF rate.

Although many researchers have reported factors that could predict extubation success (ES) or the expectation model of EF, they have not been widely used because of their lack of accuracy [7–10]. To date, factors that affect EF in preterm infants have been widely reported; however, reports are lacking on the factors that affect EF in preterm infants of a similar gestational age and birth weight. Therefore, here we investigated the factors that affected ES or EF in preterm infants matched for gestational age and birth weight.

## Methods

### Patients

This retrospective study included early preterm infants born at Pusan National University Hospital between January 2017 and December 2021. Preterm infants with a gestational age of < 32 weeks who were intubated, received MV for > 24 h, and underwent attempted extubation at least once were enrolled. The exclusion criteria were as follows: 1) death before the first extubation attempt; and 2) transfer to another hospital.

The clinical team decided on intubation/re-intubation using the following criteria: 1) fraction of inspired oxygen (FiO_2_) on noninvasive respiratory support (NRS) ≥ 0.2 above the initial FiO_2_ value required to maintain a peripheral oxygen saturation of 90% [11]; 2) Silverman Anderson retraction score > 4, indicating respiratory failure [12]; 3) repetitive apneas within 3 h; 4) insufficient respiratory drive; and 5) respiratory acidosis with a partial pressure of CO_2_ (PaCO_2_) > 65 mmHg and pH < 7.2. The decision to extubate was based on the following criteria: 1) hemodynamic stability; 2) sufficient respiratory drive; 3) oxygen saturation > 90% with a mean airway pressure (MAP) < 8 cmH_2_O and FiO_2_ < 0.3; and 4) absence of any other pathologic conditions, such as shock and sepsis.

After extubation, all patients received nasal continuous positive airway pressure (nCPAP) or high-flow nasal cannula (HFNC) as NRS. According to our unit’s policy and available equipment, patients whose corrected age was < 28 weeks’ gestation or whose body weight was < 1,500 g at the time of the first extubation were placed on nCPAP: otherwise, they were administered HFNC. During nCPAP, the FiO_2_, positive end-expiratory pressure, and flow rate were set at 0.3, 5-6cmH_2_O and 5-8L/min, respectively. The FiO_2_ and flow rate of the HFNC were started at 0.3 and 2 L/kg/min, respectively.

### Data collection

We matched ES patients for gestational age and birth weight to EF patients. Demographic, neonatal, maternal, and ventilation-associated data and clinical outcomes were collected. Demographic and neonatal factors included gestational age, sex, birth weight, 1- and 5-min Apgar scores, use of inotropic agents within 7 days of age, administration and number of pulmonary surfactants administered through an endotracheal tube, presence of patent ductus arteriosus (PDA), and persistent pulmonary hypertension of the newborn. Ventilation-associated factors were: respiratory severity score (RSS) at birth, 1 day, 3 days, 1 week, 2 weeks, 3 weeks, 4 weeks of age, and extubation; corrected age; pH; PCO_2_; body weight at the first extubation attempt; and oxygen supply after the first extubation. Regarding the EF group, we added the reason for re-intubation, extubation duration after the first extubation, hospital day at the second extubation attempt, and the total number of extubation attempts.

Clinical outcomes were collected, including BPD severity, age at full enteral feeding (> 100 mL/kg/day), duration of oxygen supply, duration of hospital stay, corrected age, body weight and percentile at discharge, tracheostomy, need for home oxygen at discharge, and other co-morbidities, such as IVH and retinopathy of prematurity (ROP).

Maternal data included age at delivery, presence of pathologic chorioamnionitis, and the use and number of antenatal steroids.

### Definitions

Extubation success was defined as survival for ≥ 7 days without the need for intubation, whereas EF was defined as the need for intubation within 7 days of extubation. The chief clinician decided on the initial intubation, timing of extubation, method of post-extubation respiratory support, and need for re-intubation. If high-frequency ventilation was used, it was converted to conventional MV mode before extubation. The percentage body weight was calculated based on Fenton (2013) [13]. Intrauterine growth retardation was defined as < 10^th^ birth weight percentile for gestational age.

A pediatric cardiologist diagnosed the patients with PDA, pulmonary hypertension, and/or other congenital heart diseases using echocardiography. Persistent pulmonary hypertension of newborns was defined as requiring treatment with nitrogen monoxide after confirmation of a bidirectional shunt through the ductus arteriosus or patent foramen ovale, tricuspid regurgitation, or flattening of the interventricular septum on echocardiography. IVH was diagnosed by a pediatric radiologist as grade 3–4 germinal matrix hemorrhage on cranial ultrasonography. PDA, which showed hemodynamic instability, such as severe pulmonary hemorrhage, severe metabolic acidosis, and increased ventilator settings, was treated with ibuprofen or surgery. Patients with contraindications to or who failed ibuprofen treatment underwent surgery. RSS was defined as the MAP multiplied by FiO2, ROP was defined as stage 3 with a plus sign, and stage 4 or 5 of ROP by a pediatric ophthalmologist. The diagnosis and severity of BPD were based on the Jobe-Bancalari criteria [14]. If the oxygen saturation (SpO_2_) was persistently < 90% despite an FiO_2_ > 0.4 and a chest radiograph showed diffuse ground-glass opacity, air bronchogram, or a total white-out, respiratory distress syndrome (RDS) was diagnosed [15]. Pulmonary surfactants were administered when RDS was confirmed. Systemic steroid use for weaning from MV was chosen when patients had no central catheter and no signs of infection. Per protocol, a total of 1.1 mg of dexamethasone was administered intravenously or orally over 10 days.

Antenatal steroid was defined as dexamethasone 5 mg twice daily for 2 days at a gestational age of > 24 weeks. Steroid administration not completed 24 h before delivery was considered not having used antenatal steroids.

### Statistical analysis

We matched patients in 1:1 ratio from the ES and EF groups using the propensity score matching method. Using logistic regression analysis, we calculated the propensity score using gestational age and birth weight as covariates. However, we excluded five patients in the ES group because they showed a gestational age and birth weight that was more than two standard deviations above the average gestational age and birth weight of the EF group. Finally, we included 19 patients in the ES group and 24 in the EF group.

All statistical analyses were performed using SPSS version 22 (IBM Corp., Armonk, NY, USA). When comparing the two variables, continuous and non-continuous variables were analyzed using the t-test and chi-squared test, respectively. In addition, the area under the receiver operating characteristic (ROC) curve analysis was performed. Finally, the optimal RSS cutoff values at 1 and 4 weeks of age were evaluated based on the ROC.

## Results

**Fig. 1:**
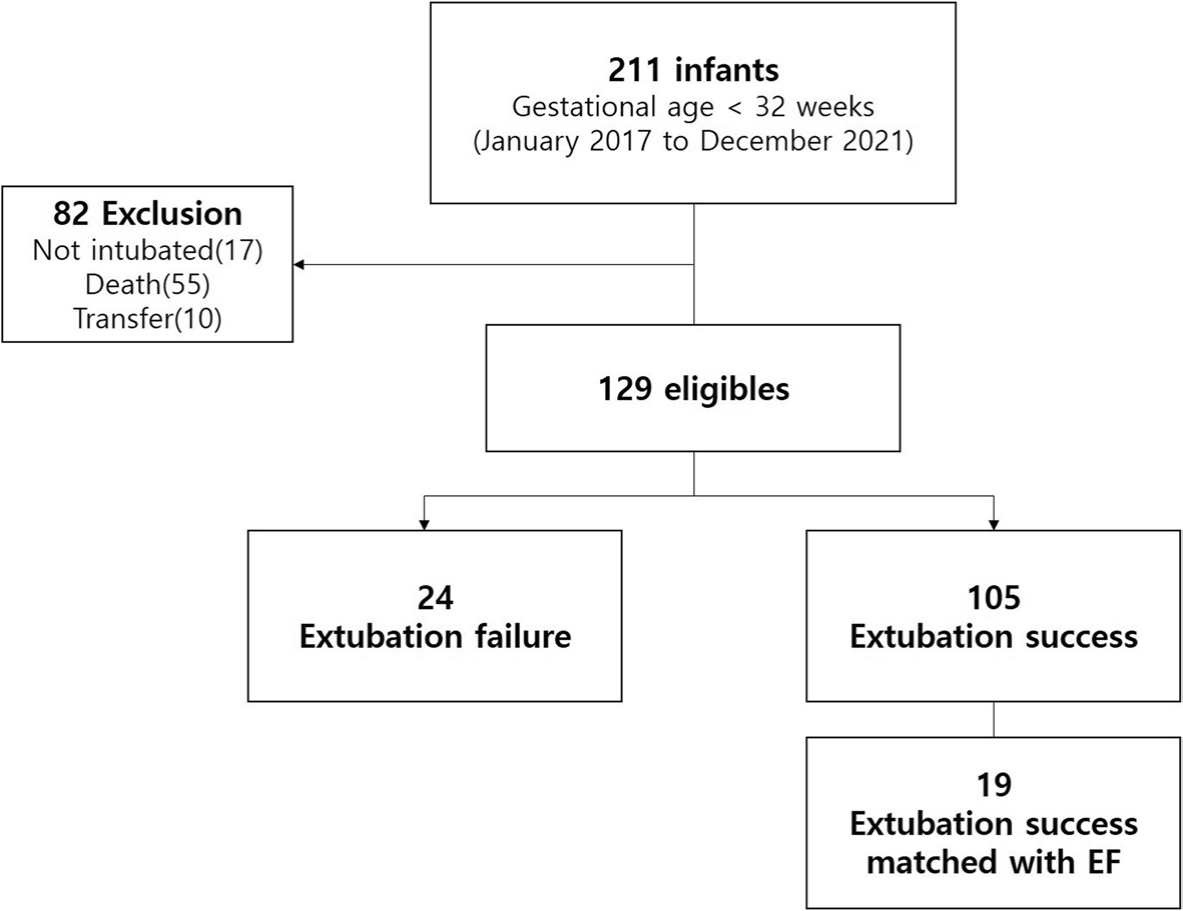
Flow diagram of the study population

Of the 211 infants born at < 32 weeks of gestational age at our hospital during the study period, 194 were intubated within 24 h after birth. A total of 55 infants died before the first attempt at extubation, and 10 were transferred to another hospital. Finally, 129 infants remained; of them, 24 in whom extubation failed at least once were included in the EF group. The EF rate was 18.6% (24/129). Of the 105 patients in whom extubation was successful, 19 matched with the EF group for gestational age and birth weight were included in the ES group (Fig. 1).

**Fig. 2:**
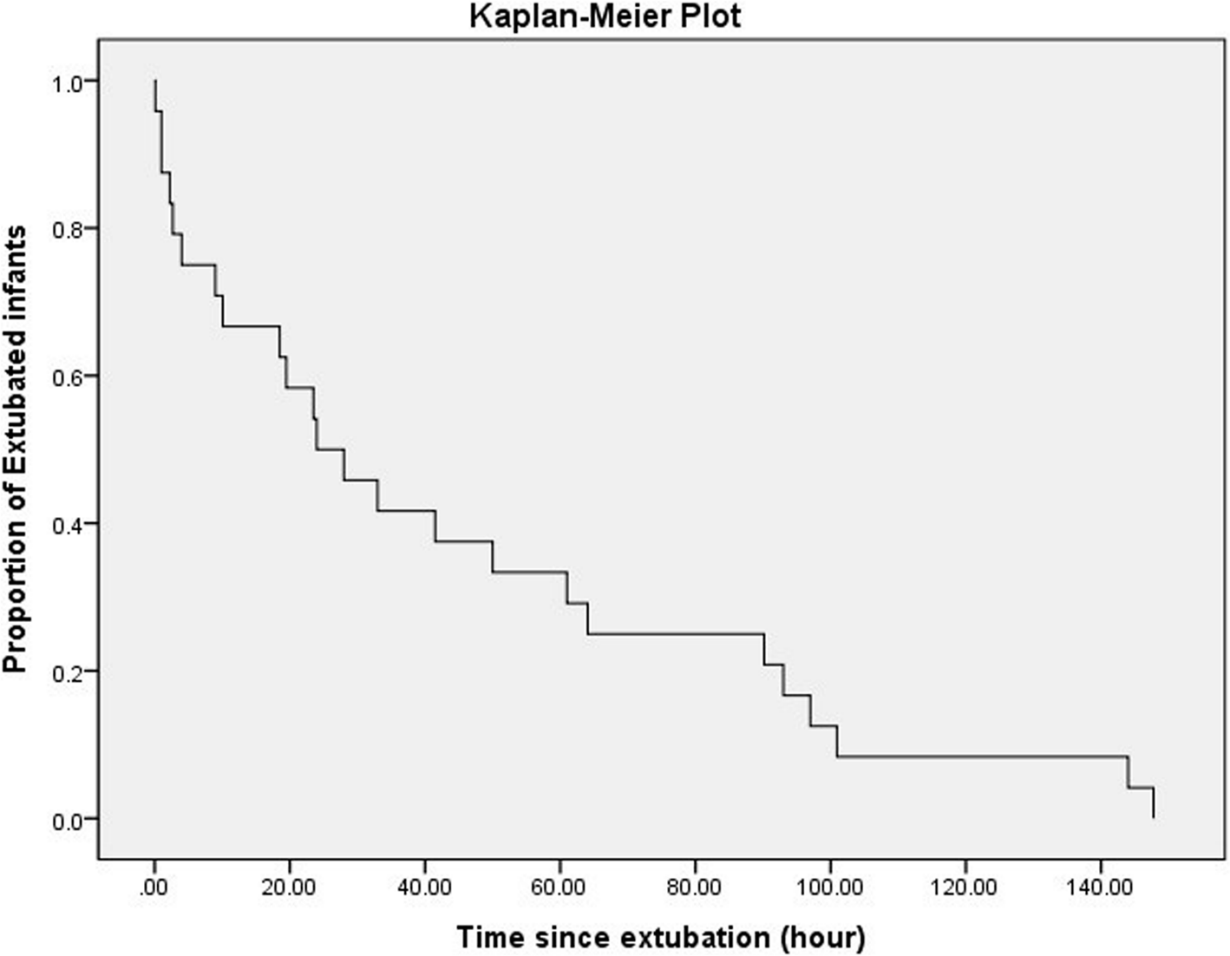
Kaplan–Meier plot showing the proportion of infants remaining extubated within 120 h. Among those in whom extubation failed within 120 h, 79.9% underwent re-intubation within 90.2 h

The most common reason for re-intubation was increased work of breathing (66.7%), such as chest retraction and tachypnea, to maintain an SpO_2_ > 90%. The average duration of the noninvasive oxygen supply was 44.2 h. Additionally, 79.9% of the extubated patients were re-intubated within 90.2 h (Fig. 2). Re-extubation was performed an average of 50.1 days after hospitalization and 24.6 days after re-intubation. On average, 2.42 extubation attempts were made in the EF group; up to five extubation attempts were made (Table 1).

**Table 1:** Re-intubation and second extubation in EF group

Variable	EF group (*n* = 24)
Hours remaining extubated after first extubation, hour, median (IQR)	44.4(0.08–147.75)
**Reason for re-intubation**	
Apnea, n (%)	8(33.3%)
Work of breathing, n (%)	16(66.7%)
Hospitalization at second extubation, days, median (IQR)	50.1(13–110)
Total number of extubation, median (IQR)	2.42(2–5)

Values are shown as n (%) or median (interquartile range)

The demographic, neonatal, maternal, and ventilation-associated data for the EF and ES groups are presented in Table 2. Demographic, neonatal, maternal, and ventilation-associated data for the EF and ES groups before matching are presented in the supplementary material. In the ES group, there was less use of inotropes within 7 days of life, a lower RSS at 1 and 4 weeks of age, and a faster time to reach full feeding. Furthermore, antenatal steroid use was greater in the ES group.

**Table 2:** Demographic, neonatal, maternal, and ventilation-associated data for EF and ES groups

Variable	Extubation failure (*n* = 24)	Extubation success (*n* = 19)	*P* value
**Characteristics of neonate**			
Male sex, n (%)	18 (75%)	9 (47.4%)	0.063
Gestational age, weeks, median (SD)	27.7 (1.4)	27.6 (1.3)	0.873
Birth weight, grams, median (SD)	988.6 (257)	1070.9 (206)	0.263
Intrauterine growth retardation, n (%)	4 (16.7%)	1 (5.3%)	0.247
Apgar score at 1 min, median (IQR)	3.79 (2–5)	3.74 (2–5)	0.835
Apgar score at 5 min, median (IQR)	5.71 (4–7)	5.84 (3–8)	0.642
Surfactant use, median (IQR)	1.42 (1–3)	1.42 (1–3)	0.982
Inotropic use within 7 days of age, n (%)	22 (91.7%)	12 (63.2%)	0.022
PDA, n (%)	14 (58.3%)	8 (42.2%)	0.396
PPHN, n (%)	4 (16.7%)	3 (15.8%)	0.938
HD of full feeding, days, median (IQR)	29.7 (6–70)	18.7 (4–47)	0.020
**RSS**			
Birth, median (IQR)	3.29 (1.55–15.00)	2.69 (1.75–7.20)	0.373
1 day of age, median (IQR)	2.87 (1.40–15.00)	2.52 (1.49–6.60)	0.606
3 days of age, median (IQR)	2.67 (1.27–10.50)	1.97 (0–3.96)	0.175
1 week of age, median (IQR)	2.50 (1.42–6.00)	1.72 (0–3.60)	0.026
2 weeks of age, median (IQR)	2.51 (1.38–4.50)	1.90 (0–4.40)	0.110
3 weeks of age, median (IQR)	2.54 (1.48–4.00)	2.15 (0–4.94)	0.359
4 weeks of age, median (IQR)	2.92 (0–5.28)	1.73 (0–4.41)	0.010
**Pre-extubation**			
HD, days, median (IQR)	25.6 (3–81)	29.0 (1–86)	0.636
Corrected age, weeks, median (IQR)	31.3 (26–39)	31.8 (27.6–37.7)	0.625
Body weight, gram, median (IQR)	1374.6 (668–3042)	1474.6 (914–2605)	0.535
Use of systemic steroid for extubation, n (%)	23(95.8%)	15 (78.9%)	0.086
pH, median (IQR)	7.32 (7.07–7.55)	7.37 (7.16–7.54)	0.076
PCO_2_, median (IQR)	42.0 (19.2–59.6)	39.6 (18.3–53.1)	0.564
RSS, median (IQR)	1.88 (1.47–2.39)	1.79 (1.30–2.38)	0.322
**Oxygen supply after extubation**			
Nasal CPAP, n (%)	11 (45.8%)	8 (42.1%)	0.357
HFNC, n (%)	13 (54.2%)	11 (57.9%)	
**Outcome**			
BPD severity			0.018
None	0	0	
Mild, n (%)	6 (25%)	10 (52.6%)	
Moderate, n (%)	4 (16.7%)	6 (31.6%)	
Severe, n (%)	14 (58.3%)	3 (15.8%)	
IVH, n (%)	2 (8.3%)	2 (10.5%)	0.806
ROP. n (%)	12 (50%)	8 (42.1%)	0.606
Duration of oxygen supply, days, median (IQR)	92.9 (48–275)	66.5 (27–110)	0.042
Tracheostomy, n (%)	1 (4.2%)	0	0.368
Home oxygen, n (%)	3 (12.5%)	0	0.110
HD at discharge, days, median (IQR)	103.4 (57–254)	84.1 (44–122)	0.074
Corrected age at discharge, weeks, median (IQR)	42.5 (37–64)	39.6 (35.9–45.0)	0.043
Body weight at discharge, grams, median (IQR)	3209.2 (2200–6150)	2820.0 (2200–3510)	0.080
**Maternal and prenatal care information**			
Age, median (IQR)	33.8 (26–41)	34.6 (25–41)	0.553
Chorioamnionitis, n (%)	10 (41.7%)	10 (52.6%)	0.486
PROM, n (%)	7 (29.2%)	11 (57.9%)	0.060
Antenatal steroid, median (IQR)	0.71 (0–1)	0.95 (0–1)	0.047

Values are shown as n (%) or median (interquartile range)

*BPD* Bronchopulmonary dysplasia, *CPAP* Continuous positive airway pressure, *HD* Hospital day, *HFNC* High-flow nasal cannula, *IVH* Intraventricular hemorrhage, *PDA* Patent ductus arteriosus, *PPHN* Pulmonary hypertension of newborn, *PROM* Premature rupture of membrane, *ROP* Retinopathy of prematurity, *RSS* Respiratory severity score

**Fig. 3:**
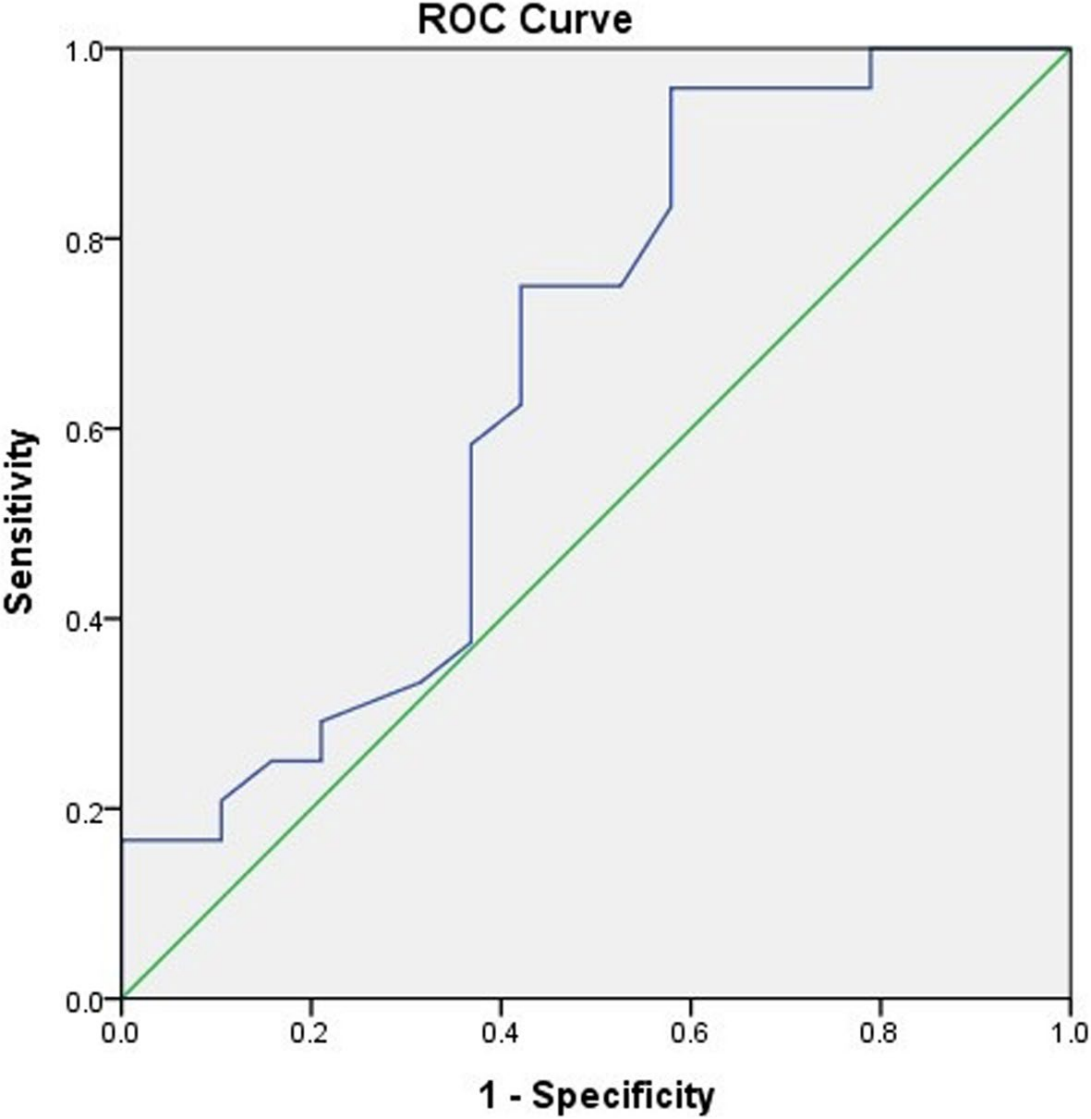
Receiver operating characteristic curve of respiratory severity score at 1 week predicted the risk of extubation failure. Area under the curve = 0.659

**Fig. 4:**
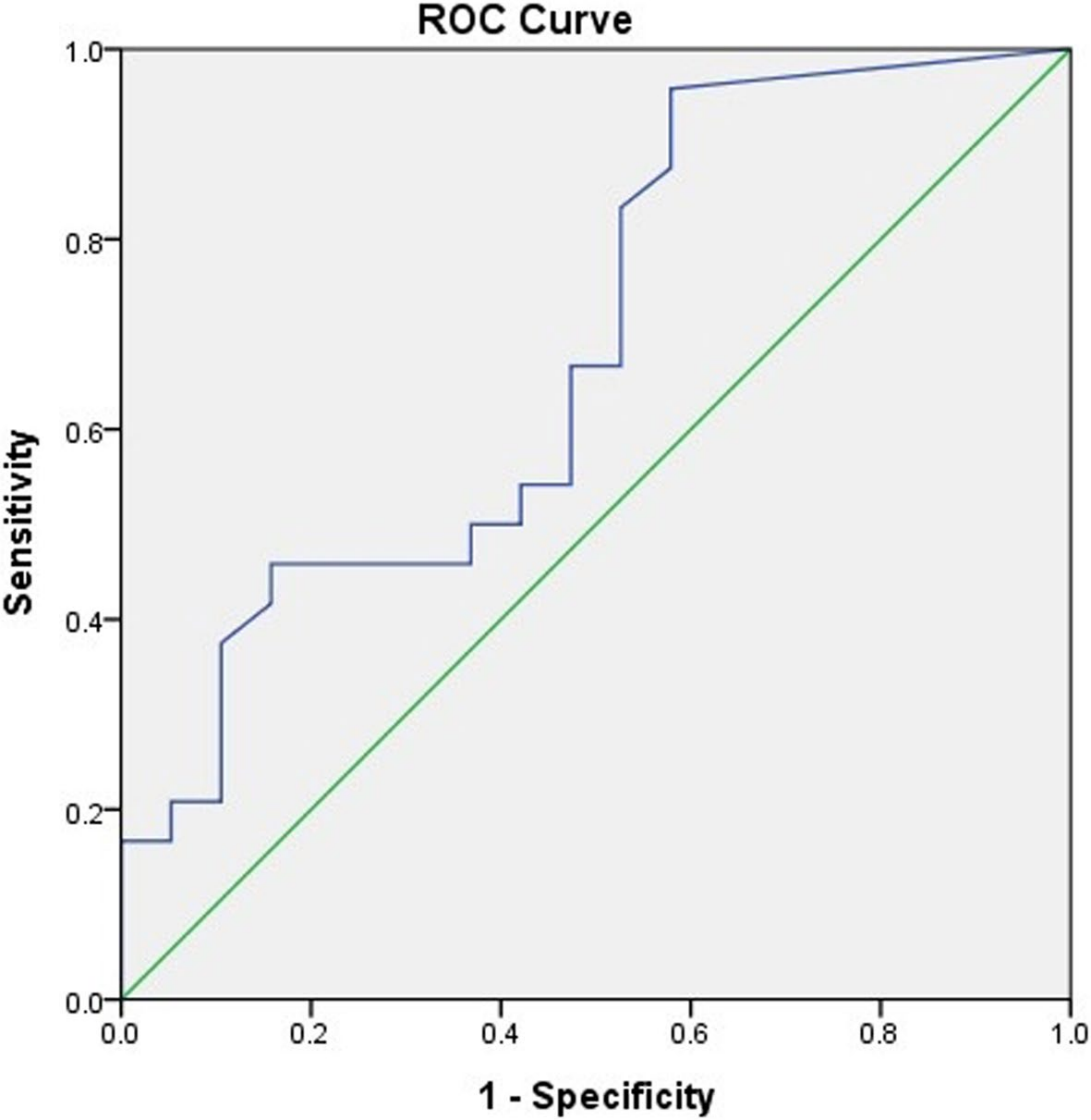
Receiver operating characteristics curve of respiratory severity score at 4 weeks predicted the risk of extubation failure. Area under the curve = 0.684

The ROC curve was derived to calculate the cutoff value, sensitivity, and specificity for RSS at 1 and 4 weeks after birth. The area under the ROC curve was 0.66 and 0.68, respectively (Figs. 3 and 4). The cutoff value, sensitivity, and specificity of RSS at 1 week of age were 1.98, 0.71, and 0.42, respectively, while those at 4 weeks of age were 2.22, 0.67, and 0.47, respectively.

Concerning clinical outcomes*,* the EF group had a higher BPD severity, longer oxygen supply duration, and higher corrected age at discharge*.*

## Discussion

The EF rate in preterm infants is relatively high, and those with EF are more likely to have long-term complications such as ventilator-associated pneumonia and BPD [2–5]. Studies that analyzed the factors affecting EF in preterm infants reported that a younger gestational age, lower birth weight, lower 5-min Apgar score, and lower PaCO_2_ before extubation increase EF [9, 10]. In this retrospective study, gestational age and birth weight were matched between the ES and EF groups. Moreover, no differences were noted in 5-min Apgar scores and PaCO_2_ before extubation. Rather, inotropic use within 7 days of age, delayed full enteral feeding, and high RSS at 1 week and 4 weeks of age affected EF. The EF group showed more severe BPD, a longer oxygen supply duration, and a higher corrected age at discharge, indicating similar results to those of previous studies [5, 9, 10].

Several studies have reported EF rates in preterm infants. Chawla et al. showed that the EF rate in preterm infants born at < 28 weeks was 37–50%, while Teixeira et al. reported that the EF rate of infants with a very low birth weight (< 1,500 g) was 23.3% [5, 9]. Wissam et al. reported that the cumulative rate of EF was the highest within 3–7 days after extubation. In this study, the EF rate was 12.3%, and 80% of patients with EF were re-intubated within 4 days later. The EF rate was lower than that in previous studies; however, the cumulative re-intubation rate was similar to that in the study by Wissam et al. The EF rate was low in our study because the timing of our center's first extubation was later than that in the previous study and performed at a higher postmenstrual age (PMA). First, extubation was attempted on the patients in previous studies at 2–16 days of age (27.0–29.5 weeks of PMA); however, the first extubation in this study was performed at 25–29 days of age and 31 weeks of PMA. These results are presumed similar to the low EF rates in preterm infants with a high gestational age and birth weight.

Previous studies reported that the use of inotropes in preterm infants at an early age is associated with a higher risk of death, IVH, and BPD [16–18]. In particular, hypotension treatment in early life can reportedly affect changes in cerebral blood flow, causing IVH or hydrocephalus and long-term neurodevelopmental delays. In the present study, the EF group showed greater use of inotropes within 7 days of age. Although it is unclear by which mechanisms the use of inotropes in early life affects EF, based on previous studies that reported a higher rate of severe intracranial hemorrhage in patients with EF [5], hypotension or blood pressure instability in early life can affect lung and brain maturity.

The early introduction of full enteral feeding can affect mortality and morbidity in preterm infants by enhancing micronutrient delivery, promoting intestinal development and maturation, stimulating microbiome development, reducing inflammation, and enhancing brain growth and neurodevelopment [19]. If preterm infants fail to achieve full enteral feeding, they may require total parenteral nutrition (TPN) for a longer period, which may lead to an indwelling central venous catheter for a longer period. Capasso et al. reported that a long indwelling period of the central venous catheter could cause systemic infection and that patients with EF had a higher risk of late-onset sepsis [20]. It has also been reported that long-term TPN can produce peroxide by the interaction between nutrients, which can cause alveolar loss and slow alveolar development, resulting in a high risk of BPD [21]. For these reasons, early full enteral feeding could be considered a factor that increases ES by affecting lung maturity.

The respiratory severity score is a respiratory index that can evaluate the severity of oxygenation failure and pulmonary status in patients receiving MV, which is calculated by multiplying MAP and FiO_2_, and can be used as an alternative to the oxygenation index, which requires invasive arterial catheterization [22]. EF patients showed a higher RSS on the first extubation attempt than ES patients [8, 23, 24]. However, this difference was not statistically significant, possibly because of the small number of patients included in our study. However, Jung et al. analyzed RSS in preterm infants born at < 28 weeks’ gestation at 2 days of age and 1, 2, 3, and 4 weeks of age and found that a high RSS at 2, 3, and 4 weeks proportionally increased the risk of severe BPD and death [25]. Moreover, Manish et al. reported that an RSS > 6 at 30 days of age is associated with prolonged MV [25, 26]. This is similar to the results of the present study, in which a higher RSS at 4 weeks increased the risk of EF. A high RSS/kg at 2 days of age increased the risk of pulmonary hypertension, which increased the prevalence of severe BPD. This is probably due to unfavorable acute-stage pulmonary mechanics and an underdeveloped alveolar structure, leading to a more severe pattern of RDS and associated with the risk of pulmonary hypertension [27, 28]. RSS in early life, such as RSS at 1 week of age in our study, reflects unfavorable pulmonary mechanics that could increase the risk of EF.

According to our study, the EF group tended to have higher BPD severity. This is consistent with the results of previous studies reporting that EF itself is an independent risk factor for BPD [5, 9, 10]. Severe BPD is defined as a longer duration of respiratory support [14], which can prolong the hospital stay and increase the corrected age at discharge.

In this study, all enrolled patients were provided NRS, either nCPAP or HFNC. There was no significant intergroup difference according to NRS mode in this study because of the similar ratio of nCPAP and HFNC use. There is no definite guideline regarding which mode is better for post-extubation NRS. A Cochrane review reported that the efficacy of HFNC for respiratory support after extubation in extremely preterm infants (gestational age < 28 weeks) was unclear because of the lack of sufficient data. However, there was no difference in the rates of EF or re-intubation in preterm infants treated with HFNC versus CPAP [29]. A systematic review reported that HFNC was non-inferior to other post-extubation modalities and resulted in a lower incidence of nasal injury versus CPAP. However, synchronized noninvasive intermittent positive pressure ventilation (SNIPPV) is the most efficacious NRS modality for preventing EF, as it results in a lower incidence of air leaks and BPD [30]. Therefore, SNIPPV may be the first choice for post-extubation NRS. However, SNIPPV was not applied to the patients in this study as the required equipment was not available. This is a limitation of this study.

This study has some additional limitations, including its small sample size and the limitations inherent to retrospective reviews. Moreover, there may have been selection bias in the matched patient collection. Further well-designed prospective studies with larger numbers of cases are needed. Despite these limitations, the gestational age and birth weights were similar between groups, eliminating their effects. This differs from the results of previous studies.

## Conclusions

In summary, using inotropes within 7 days of age, unsuccessful enteral feeding, or a higher RSS at 1 week and 4 weeks of age could increase the risk of EF. EF can cause adverse short-term outcomes such as greater BPD severity and longer duration of hospital stay. Therefore, extubation in very early preterm infants should be attempted carefully, and the use of inotropes, feeding, and RSS at 1 week of age could contribute to ES.

## Supplementary Information

**Additional file 1:** **Table 1.** Demographic, neonatal, maternal, andventilation-associated data by study group before matching. 

## Data Availability

The dataset supporting the conclusion of this article is included within the article.

## Abbreviations

BPD: Bronchopulmonary dysplasia
CPAP: Continuous positive airway pressure
EF: Extubation failure
ES: Extubation success
FiO_2_: Fraction of inspired oxygen
HFNC: High-flow nasal cannula
IVH: Intraventricular hemorrhage
MAP: Mean airway pressure
MV: Mechanical ventilation
NRS: Noninvasive respiratory support
PDA: Patent ductus arteriosus
RDS: Respiratory distress syndrome
ROC: Receiver operating characteristic
ROP: Retinopathy of prematurity
RSS: Respiratory severity score
SNIPPV: Synchronized noninvasive intermittent positive pressure ventilation
TPN: Total parenteral nutrition

## References

[1] Stoll BJ, Hansen NI, Bell EF, Walsh MC, Carlo WA, Shankaran S (2015). Trends in care practices, morbidity, and mortality of extremely preterm neonates, 1993–2012. JAMA.

[2] Miller JD, Carlo WA (2008). Pulmonary complications of mechanical ventilation in neonates. Clin Perinatol.

[3] Jensen EA, DeMauro SB, Kornhauser M, Aghai ZH, Greenspan JS, Dysart KC (2015). Effects of multiple ventilation courses and duration of mechanical ventilation on respiratory outcomes in extremely low-birth-weight infants. JAMA Pediatr.

[4] Giaccone A, Jensen E, Davis P, Schmidt B (2014). Definitions of extubation success in very premature infants: a systematic review. Arch Dis Child Fetal Neonatal Ed.

[5] Chawla S, Natarajan G, Shankaran S, Carper B, Brion LP, Keszler M (2017). Markers of successful extubation in extremely preterm infants, and morbidity after failed extubation. J Pediatr.

[6] Sawyer T, Foglia E, Hatch LD, Moussa A, Ades A, Johnston L (2017). Improving neonatal intubation safety: a journey of a thousand miles. J Neonatal Perinatal Med.

[7] Cheng Z, Dong Z, Zhao Q, Zhang J, Han S, Gong J (2021). A prediction model of extubation failure risk in preterm infants. Front Pediatr.

[8] Gupta D, Greenberg RG, Sharma A, Natarajan G, Cotten M, Thomas R (2019). A predictive model for extubation readiness in extremely preterm infants. J Perinatol.

[9] Teixeira RF, Costa CM, Abreu CMD, Lessa CA, Carvalho AC, Kassar SB (2021). Factors associated with extubation failure in very low birth weight infants: a cohort study in the northeast Brazil. J Perinat Med.

[10] Manley BJ, Doyle LW, Owen LS, Davis PG (2016). Extubating extremely preterm infants: predictors of success and outcomes following failure. J Pediatr.

[11] Manley BJ, Owen LS, Doyle LW, Andersen CC, Cartwright DW, Pritchard MA (2013). High-flow nasal cannulae in very preterm infants after extubation. N Engl J Med.

[12] Ekhaguere OA, Okonkwo IR, Batra M, Hedstrom AB (2022). Respiratory distress syndrome management in resource limited settings—Current evidence and opportunities in 2022. Front Pediatr.

[13] Chou JH, Roumiantsev S, Singh R (2020). PediTools electronic growth chart calculators: applications in clinical care, research, and quality improvement. J Med Internet Res.

[14] Jobe AH (2011). The new bronchopulmonary dysplasia. Curr Opin Pediatr.

[15] Walsh BK, Daigle B, DiBlasi RM, Restrepo RD (2013). AARC clinical practice guideline. Surfactant replacement therapy: 2013. Respir Care.

[16] Luo N, Jiang S, McNamara PJ, Li X, Guo Y, Wang Y (2021). Cardiovascular pharmacological support among preterm infants in Chinese referral center neonatal intensive care units. Front Pediatr.

[17] Faust K, Härtel C, Preuß M, Rabe H, Roll C, Emeis M (2015). Short-term outcome of very-low-birthweight infants with arterial hypotension in the first 24 h of life. Arch Dis Child Fetal Neonatal Ed.

[18] Verma RP, Dasnadi S, Zhao Y, Chen HH (2019). Complications associated with the current sequential pharmacological management of early postnatal hypotension in extremely premature infants. Proc (Bayl Univ Med Cent).

[19] Thoene M, Anderson-Berry A (2021). Early enteral feeding in preterm infants: a narrative review of the nutritional, metabolic, and developmental benefits. Nutrients.

[20] Capasso L, Borrelli AC, Cerullo J, Caiazzo MA, Coppola C, Palma M (2022). Reducing post-extubation failure rates in very preterm infants: is BiPAP better than CPAP?. J Matern Fetal Neonatal Med.

[21] Lavoie J-C, Chessex P (2019). Parenteral nutrition and oxidant stress in the newborn: a narrative review. Free Radical Biol Med.

[22] Iyer NP, Mhanna MJ (2013). Non-invasively derived respiratory severity score and oxygenation index in ventilated newborn infants. Pediatr Pulmonol.

[23] Mhanna MJ, Iyer NP, Piraino S, Jain M (2017). Respiratory severity score and extubation readiness in very low birth weight infants. Pediatr Neonatol.

[24] Dursun M, Zubarioglu AU, Bulbul A (2021). Relationship between the respiratory severity score and extubation failure in very-low-birth-weight premature infants. Sisli Etfal Hastan Tip Bul.

[25] Jung YH, Jang J, Kim HS, Shin SH, Choi CW, Kim EK (2019). Respiratory severity score as a predictive factor for severe bronchopulmonary dysplasia or death in extremely preterm infants. BMC Pediatr.

[26] Malkar MB, Gardner WP, Mandy GT, Stenger MR, Nelin LD, Shepherd EG (2015). Respiratory severity score on day of life 30 is predictive of mortality and the length of mechanical ventilation in premature infants with protracted ventilation. Pediatr Pulmonol.

[27] Seo YM, Yum SK, Sung IK (2020). Respiratory severity score with regard to birthweight during the early days of life for predicting pulmonary hypertension in preterm infants. J Trop Pediatr.

[28] Dani C, Corsini I, Cangemi J, Vangi V, Pratesi S (2017). Nitric oxide for the treatment of preterm infants with severe RDS and pulmonary hypertension. Pediatr Pulmonol.

[29] Wilkinson D, Andersen C, O'Donnell CPF, De Paoli AG, Manley BJ (2016). High flow nasal cannula for respiratory support in preterm infants. Cochrane Database Syst Rev.

[30] Ramaswamy VV, Bandyopadhyay T, Nanda D, Bandiya P, More K, Oommen VI (2020). Efficacy of noninvasive respiratory support modes as postextubation respiratory support in preterm neonates: a systematic review and network meta-analysis. Pediatr Pulmonol.

