# Supplementary material for: Risk factors and clinical outcomes of extubation failure in very early preterm infants: a single-center cohort study
Source: BMC Pediatr. 2023 Jan 21;23:36. doi: 10.1186/s12887-023-03833-5 (PMC9863082; doi:10.1186/s12887-023-03833-5)
Supplement: Supplementary file 1 — Additional file 1: Table 1. Demographic, neonatal, maternal, andventilation-associated data by study group before matching. [file 12887_2023_3833_MOESM1_ESM.pdf]

Table 1 Demographic, neonatal, maternal, and ventilation-associated data by study group before matching

| Variable | Extubation failure (n=24) | Extubation success (n=105) | P value |
|---|---|---|---|
| **Characteristics of neonate** | | | |
| Male sex, n (%) | 18 (75%) | 58 (55.2%) | 0.082 |
| Gestational age, weeks, median (SD) | 27.7 (1.4) | 29.8 (1.7) | 0.000 |
| Birth weight, grams, median (SD) | 988.6 (257) | 1388.2 (358) | 0.000 |
| Intrauterine growth retardation, n (%) | 4 (16.7%) | 4 (3.8%) | 0.034 |
| Apgar score at 1 min, median (IQR) | 3.79 (2-5) | 4.54 (1-8) | 0.006 |
| Apgar score at 5 min, median (IQR) | 5.71 (4-7) | 6.50 (3-9) | 0.001 |
| Surfactant use, median (IQR) | 1.42 (1-3) | 1.24 (0-3) | 0.208 |
| Inotropic use within 7 days of age, n (%) | 22 (91.7%) | 55 (52.3%) | 0.000 |
| PDA, n (%) | 14 (58.3%) | 27 (25.7%) | 0.025 |
| PPHN, n (%) | 4 (16.7%) | 6 (5.7%) | 0.115 |
| HD of full feeding, days, median (IQR) | 29.7 (6-70) | 14.2 (3-66) | 0.000 |
| **RSS** | | | |
| Birth, median (IQR) | 3.29 (1.55-15.00) | 2.50 (1.50-7.20) | 0.167 |
| 1 day of age, median (IQR) | 2.87 (1.40-15.00) | 2.30 (0-16.0) | 0.208 |
| 3 days of age, median (IQR) | 2.67 (1.27-10.50) | 1.90 (0-16.0) | 0.104 |

| | | | |
|---|---|---|---|
| 1 week of age, median (IQR) | 2.50 (1.42-6.00) | 1.13 (0-5.68) | 0.000 |
| 2 weeks of age, median (IQR) | 2.51 (1.38-4.50) | 0.88 (0-9.60) | 0.000 |
| 3 weeks of age, median (IQR) | 2.54 (1.48-4.00) | 1.47 (0-5.70) | 0.000 |
| 4 weeks of age, median (IQR) | 2.92 (0-5.28) | 0.74 (0-9.0) | 0.000 |
| **Pre-extubation** | | | |
| HD, days, median (IQR) | 25.6 (3-81) | 13.60 (0-106) | 0.012 |
| Corrected age, weeks, median (IQR) | 31.3 (26-39) | 31.7 (27.6-41.0) | 0.536 |
| Body weight, gram, median (IQR) | 1374.6 (668-3042) | 1526.8 (900-2605) | 0.240 |
| Use for the systemic steroid use for extubation, n (%) | 23 (95.8%) | 22 (21.0%) | 0.000 |
| pH, median (IQR) | 7.32 (7.07-7.55) | 7.37 (7.16-7.58) | 0.017 |
| $PCO_2$, median (IQR) | 42.0 (19.2-59.6) | 36.1 (18.0-61.6) | 0.018 |
| RSS, median (IQR) | 1.88 (1.47-2.39) | 1.77 (1.24-2.80) | 0.094 |
| **Oxygen supply after extubation** | | | |
| Nasal CPAP, n (%) | 11 (45.8%) | 21 (20%) | 0.025 |
| HFNC, n (%) | 13 (54.2%) | 84 (80%) | |
| **Outcome** | | | |
| BPD severity | | | 0.000 |
| None | 0 | 47 (44.8%) | |
| Mild, n (%) | 6 (25%) | 29 (27.6%) | |
| Moderate, n (%)e | 4 (16.7%) | 18 (17.1%) | |
| Severe, n (%) | 14 (58.3%) | 11 (10.5%) | |
| IVH, n (%) | 2 (8.3) | 4 (3.8%) | 0.432 |

| | | | |
|---|---|---|---|
| ROP, n (%) | 12 (50%) | 26 (24.8%) | 0.043 |
| Duration of oxygen supply, days, median (IQR) | 92.9 (48-275) | 47.0 (27-110) | 0.000 |
| Tracheostomy, n (%) | 1 (4.2%) | 0 | 0.049 |
| Home oxygen, n (%) | 3 (12.5%) | 1 (0.9%) | 0.006 |
| HD at discharge, days, median (IQR) | 103.4 (57-254) | 64.6 (31-193) | 0.000 |
| Corrected age at discharge, weeks, median (IQR) | 42.5 (37-64) | 38.6 (35.7-53.4) | 0.001 |
| Body weight at discharge, grams, median (IQR) | 3209.2 (2200-6150) | 2812.1 (2070-4350) | 0.043 |
| **Maternal and prenatal care information** | | | |
| Age, median (IQR) | 33.8 (26-41) | 34.0 (22-41) | 0.862 |
| Chorioamnionitis, n, (%) | 10 (41.7%) | 33 (31.4%) | 0.817 |
| PROM, n (%) | 7 (29.2%) | 40 (38.1%) | 0.016 |
| Antenatal steroid | 0.71 (0-1) | 0.92 (0-2) | 0.455 |

BPD, bronchopulmonary dysplasia; CPAP, continuous positive airway pressure; HD, hospital day, HFNC, high-flow nasal cannula; IVH, intraventricular hemorrhage; PDA, patent ductus arteriosus; PPHN, pulmonary hypertension of newborn; PROM, premature rupture of membrane; ROP, retinopathy of prematurity; RSS, respiratory severity score

Values are shown as n (%), mean (standard deviation), or median (interquartile range)
